# Thermal Stability of Cu-Al-Ni Shape Memory Alloy Thin Films Obtained by Nanometer Multilayer Deposition

**DOI:** 10.3390/nano13182605

**Published:** 2023-09-21

**Authors:** Jose F. Gómez-Cortés, María L. Nó, Andrey Chuvilin, Isabel Ruiz-Larrea, Jose M. San Juan

**Affiliations:** 1 Departamento de Física, Facultad de Ciencia y Tecnología, Universidad del País Vasco (UPV/EHU), Apto. 644, 48080 Bilbao, Spain; josefernando.gomez@ehu.eus (J.F.G.-C.); maria.no@ehu.es (M.L.N.); isabel.ruiz@ehu.es (I.R.-L.); 2 CIC NanoGUNE BRTA, Tolosa Hiribidea 76, 20018 Donostia-San Sebastian, Spain; a.chuvilin@nanogune.eu; 3 IKERBASQUE, Basque Foundation of Science, Plaza Euskadi 5, 48009 Bilbao, Spain

**Keywords:** shape memory alloys (SMA), Cu-Al-Ni, e-beam evaporation, thin films, in situ TEM, nanoprecipitates

## Abstract

Cu-Al-Ni is a high-temperature shape memory alloy (HTSMA) with exceptional thermomechanical properties, making it an ideal active material for engineering new technologies able to operate at temperatures up to 200 °C. Recent studies revealed that these alloys exhibit a robust superelastic behavior at the nanometer scale, making them excellent candidates for developing a new generation of micro-/nano-electromechanical systems (MEMS/NEMS). The very large-scale integration (VLSI) technologies used in microelectronics are based on thin films. In the present work, 1 μm thickness thin films of 84.1Cu-12.4 Al-3.5Ni (wt.%) were obtained by solid-state diffusion from a multilayer system deposited on SiNx (200 nm)/Si substrates by e-beam evaporation. With the aim of evaluating the thermal stability of such HTSMA thin films, heating experiments were performed in situ inside the transmission electron microscope to identify the temperature at which the material was decomposed by precipitation. Their microstructure, compositional analysis, and phase identification were characterized by scanning and transmission electron microscopy equipped with energy dispersive X-ray spectrometers. The nucleation and growth of two stable phases, Cu-Al-rich alpha phase and Ni-Al-rich intermetallic, were identified during in situ heating TEM experiments between 280 and 450 °C. These findings show that the used production method produces an HTSMA with high thermal stability and paves the road for developing high-temperature MEMS/NEMS using shape memory and superelastic technologies.

## 1. Introduction

Among the different smart functional materials, shape memory alloys (SMA) offer the highest work-output per unit of weight [1], giving them some competitive advantage for working as actuators on a small scale [2]. This working capability is due to a reversible martensitic transformation between the high-temperature phase (austenite) and the low-temperature phase (martensite) through a structural phase transition involving an atomic lattice shearing, by shuffling and distortion of the austenite lattice; see the textbooks [3,4,5] for a review of the fundamental aspects of SMA. In recent years, emerging flexible technologies have incorporated thin films of SMA into the design of auxetic materials [6] and mechanical metamaterials [7,8], as well as stretchable electronics [9], origami-inspired or programmable surfaces [10,11], and in general, all technologies of thin-film flexible actuators [12]. In addition, because of the advent of miniaturization, many research efforts have focused on the characterization of SMA at the micro- and nanometer scale in order to develop active micro-/nanodevices; devices such as microgrippers [13], microswitches [14], microvalves [2], microwrappers [15], and bimorph actuators [16] have already been developed for MEMS applications; see [17,18] for a review in this field. Most of the works on this topic have been carried out on TiNi SMA [17], and thin films of this alloy have been produced by a variety of techniques. DC sputtering deposition [19,20,21,22] and RF magnetron sputtering [23,24,25] have been the most used techniques, and were recently reviewed [26,27]. However, other techniques are also used, such as co-evaporation [28], pulsed laser deposition [29] and e-beam evaporation [30]. High-throughput combinatorial thin films production [31,32] was developed to optimize the required concentration in ternary and quaternary alloys based on the TiNi system [33,34]. In contrast to the hundreds of publications on TiNi SMA thin films [17,18,26,27], only a few works have been devoted to producing and characterizing Cu-based SMA thin films [35,36,37,38,39,40,41,42,43]. Nevertheless, recent studies have shown that at the micro- and nanoscales [44,45], Cu-Al-Ni SMA exhibits shape memory and superelastic behavior superior to those of Ti-Ni, with excellent cycling behavior at these small scales [46]. These good long-term thermal and mechanical properties make this family of alloys very promising for micro-/nano-actuators, and some microdevices have already been proposed as micro-dampers [47]. In addition, TiNi functional properties are limited to temperatures below 100 °C [48,49], whereas Cu-Al-Ni SMA can be designed for high-temperature operation (up to 200 °C) [50], which represents an attractive advantage over TiNi SMA for many technological applications.

Considering that previous results have shown the remarkable shape memory and superelasticity of Cu-Al-Ni SMA at micro-/nanoscale [44,45,46,47], this work is not devoted to the study of functional properties. Instead, the approach of the present work focuses on a twofold objective. First, a robust methodology for Cu-Al-Ni SMA thin film production is developed based on the control of nanolayers from the alloying elements. This is because, as in the case of Ti-Ni, most of the Cu-based SMA thin films are obtained by the sputtering technique from a pre-alloyed target. However, the required control of the final composition becomes difficult because of the different sputtering coefficient of the aluminium with respect to copper or nickel. Then, our approach is to produce the thin film by e-beam evaporation of single elements to create a multilayer system, obtaining the target composition through further thermal treatment. Second, in order to develop high-temperature SMA, a thorough study of the thermal stability of the produced thin films is conducted through in situ heating under transmission electron microscopy (TEM). Both objectives are intended test the potential capabilities of this SMA system for further development of high-temperature small-scale actuators able to be incorporated into the new generations of MEMS, with extended temperature performances, which are required in several sectors, such as automotive, aeronautic, and aerospace.

## 2. Materials and Methods

### 2.1. Synthesis

**Figure 1:**
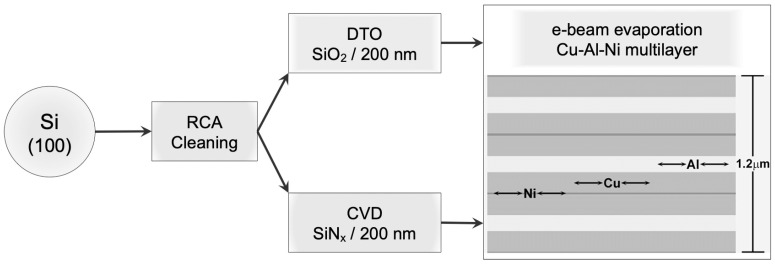
Flow diagram for obtaining a set of Cu, Al, and Ni metallic multilayers deposited by the e-beam evaporation technique on Si substrates with two types of coatings, SiO_2_ and SiN_x_, both 200 nm thick, referred to as samples CAN/SO and CAN/SN, respectively. The final step illustrates the stacking order of the metallic layers and their nominal thickness (1.2 μm).

**Figure 2:**
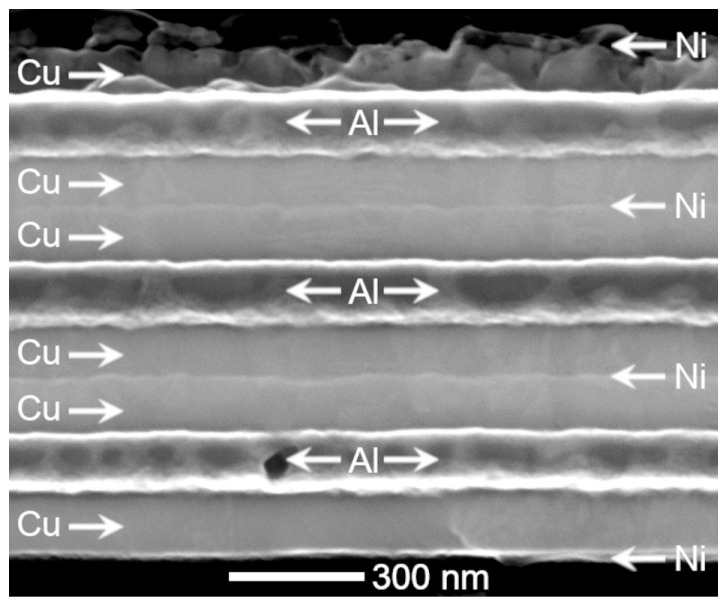
SEM image with secondary electrons (SE) of the multilayer system as deposited on the substrates. In this representative cross-sectional view, arrows and symbols indicate the chemical nature of each layer. The top layer of Cu and Ni were preferentially etched in the argon plasma.

Silicon wafers, (100)-oriented with two different coatings, were used as substrates in this work: SiN_x_ (200 nm)/Si and SiO_2_ (200 nm)/Si. These substrates were prepared using conventional techniques and fabrication procedures of the microelectronic industry. First, several wafers were cleaned by the standard RCA process [51]. Then, two wafers were submitted to a dry thermal oxidation method (DTO) to obtain a SiO_2_ layer 200 nm thick, and two other wafers were submitted to a chemical vapour deposition (CVD) treatment to obtain a SiN_x_ coating of 200 nm in thickness. Finally, Cu, Al, and Ni metallic layers were deposited over these two substrates by e-beam evaporation using a Temescal VES 2550 with planetary wafer rotation and an FDC-8000 film-deposition controller with a quartz balance. The layers were sequentially deposited at room temperature, evaporating high purity (5N) Ni, Cu, and Al targets under a high vacuum (better than 1 × 10^−4^ Pascal). The flow chart in Figure 1 summarizes the fabrication process and shows the stacking order of the deposited metallic layers. All these processes were carried out at the cleanroom facilities of the MTL at the MIT. This stacking configuration of the metallic layers was designed to prevent the contact proximity between the Ni and Al layers to avoid the likely precipitation of a highly stable Al-Ni intermetallic compound during the subsequent thermal treatment needed to obtain the desired alloy. In addition, a half layer of Ni was evaporated at the top and bottom of the stack to avoid copper oxidation during further thermal treatments. Finally, we achieved the Cu-Al-Ni multilayer system on the two substrates, SiNx/Si and SiO_2_/Si, which we here refer to as the samples CAN/SN and CAN/SO, respectively. The initial nominal composition of the alloy, 84 Cu-12 Al-4 Ni (wt.%), was selected from the expression given in [28] to obtain a very high transformation temperature Ms (martensite start) of about 360 °C. This choice aims to study the martensite stability and the precipitation processes associated with its decomposition. However, from previous deposition tests, we were aware that the quartz balance systematically under-estimated the Al composition by about 10% in mass, because of its low density, and this fact was taken into account for the design of the multilayer thickness. Consequently, the nominal composition was modified as indicated in Table 1. The nominal thickness of the whole multilayer stack was 1.2 μm, and the thickness of each metallic layer was calculated from the density; these are also indicated in Table 1 with the nominal composition of the thin film alloy. The scanning electron microscopy (SEM) image in Figure 2 reveals the cross-section of the multilayer stack as it was deposited on the substrates. This representative micrograph corroborates the presence of all metallic layers in the planned stack order and thickness ratio mentioned previously (Figure 1 and Table 1).

To obtain the Cu-based SMA alloy thin films, the multilayer systems were thermally treated at 900 °C under a high-purity (6N) Ar atmosphere for one hour, and then quenched in ice water. This heat treatment had two purposes: on the one hand, heating to promote the alloying of the three metals in the multilayer assembly by solid state diffusion, and on the other hand, quenching to 0 °C in order to obtain the metastable martensite phase at room temperature after the high-temperature austenite phase. Indeed, the stability domain of the austenite, according to the predefined concentration and the respective phase diagram [52], can be guaranteed at 900 °C.

### 2.2. Microstructural Characterization and In Situ Heating TEM

Cross-sections of the as-grown multilayers and the alloyed samples were encapsulated in epoxy resin and metallographically prepared (ground and polished until 0.05 μm alumina suspension) for their microstructural and chemical characterization. Finally, to reveal the fine microstructure, the samples were physically etched with argon plasma in a Gatan PECS 682 equipment and operated at 4 kV and 131 mA, 25 rpm sample rotation, and 75 degrees of tilt.

The cross-section microstructure and compositional analysis of the alloyed thin film were characterized by scanning electron microscopy (SEM) using a field emission gun microscope JEOL JSM-7000F equipped with an Oxford INCA Energy 350 energy dispersive X-ray spectrometer (EDX). EDX operation parameters were adjusted to limit the interaction volume to the film thickness to avoid substrate contributions. Backscattered electron (BSE) images were obtained from an accelerating voltage of 10 kV with 1 nA probe current and 10 mm working distance (WD), while the EDX spectra were acquired at 12 kV, 1 nA, and 10 mm of WD, with an acquisition time of 100 s. The quantifications were performed using K-series and pure certificated microanalysis standards (MAC Reference Standards for X-ray Microanalysis) to calibrate on-site the EDX spectra.

The in-plane (parallel to the thin film surface) microstructure characterization and its in situ evolution on heating were carried out using transmission electron microscopy (TEM, Phillips CM 200) operating at 200 kV with a double-tilt heating holder (Gatan model 652). The in situ heating experiment was carried out on an in-plane lamella extracted along the cross-section previously prepared for SEM characterization, using the conventional focused ion beam (FIB) and in situ lift-out method in a dual beam FIB/SEM equipment (FEI Helios NanoLab 600). After the in situ heating experiment, the sample was studied by TEM in a FEI Titan Cubed G2 60-300 microscope, working at 300 kV, employing the high-angle annular dark field (HAADF) and EDX (ChemiSTEM) detectors in scanning-transmission electron microscopy mode (STEM); all these equipment are from Thermo Fisher Scientific, Waltham, MA, USA. 

## 3. Results and Discussion

After the heat treatment, the CAN/SO sample experienced a compositional degradation that destroyed the thin film configuration; bulges, precipitates, and diffusion of metallic material inside the silicon substrate were observed [38]. These results deviated from the objective of obtaining a good SMA thin film, and the CAN/SO sample was discarded. Consequently, all further research was focused on the CAN/SN sample, whose results satisfied the objective, allowing the further study of in situ heating under TEM.

Initially, this CAN/SN sample was thermally treated at 900 °C to promote the alloying of the Cu, Al, and Ni layers by solid-state diffusion, and then quenched at 0 °C to obtain the martensite phase at room temperature, as previously indicated.

### 3.1. Thin Film Characterization

**Figure 3:**
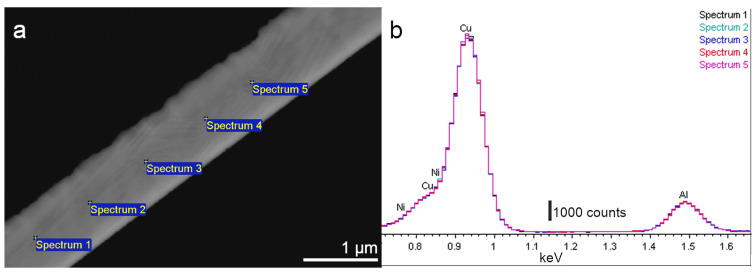
EDX analysis of the CAN/SN sample. (**a**) SEM image with Backscattered electron (BSE) of a cross-section analyzed in five points; (**b**) intensity maxima for Cu (Lα) and Al (Kα) overlap in five spectra, denoting a high compositional homogeneity.

Figure 3 shows representative results of the overall appearance of the CAN/SN sample cross-section and the EDX analysis performed along it. Figure 3a is a SEM image in BSE mode, from which can be observed a diagonal strip where five EDX spectra were taken. This strip thickness matches the expected nominal thickness of the thin film, 1.2 μm (Table 1), and the observation along the substrate confirms the homogeneous thickness of the thin film. The EDX spectra were acquired from central points along the cross-section film. The operation voltage was reduced as much as possible to avoid the silicon substrate excitation, but at least 12 kV were required to excite the Cu and Ni K-lines to proper quantification–their L lines overlap too much to be used in the quantification. Thus, with this voltage, some substrate contribution was unavoidable and a tiny amount of Si (0.1 wt.%) was systematically detected in all analysed spots. Excluding the silicon signal, the spectra only show the presence of Cu, Al, and Ni in a reasonably homogeneous distribution along the cross-section, as can be concluded from Figure 3b, where all spectra taken in different spots are overlapped in intensity. The average chemical concentration measured was 84.1Cu-12.4Al-3.5Ni (wt.%), which agreed reasonably well with the nominal concentration, as shown in Table 1, aligning with the expected difference in the Al composition associated with the quartz balance calibration. These results confirm that a homogenous thin film, in both thickness and composition, was obtained from a Cu-Al-Ni multilayer stack via thermal treatment. Once the overall homogeneity in thickness and composition was checked, the following task was focused on the microstructure and phase characterization. Considering what happened with the discarded sample (CAN/SO), it is essential to mention that the silicon nitride coating was demonstrated to be an excellent inert diffusion barrier.

**Figure 4:**
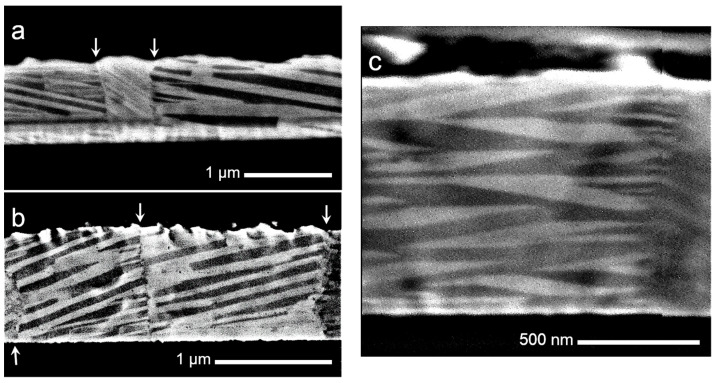
SEM images in BSE mode, exhibiting the characteristic cross-section microstructure in the CAN/SN sample. (**a**,**b**) Martensite variants inside grains cross the entire cross-section; arrows point out the grain boundaries spanning the entire thickness of the film; (**c**) self-accommodating microstructure of martensites in the thin film of SMA.

Figure 4 exhibits three BSE micrographs at different magnifications to show the microstructure characteristics observed along the thin film’s cross-section. In Figure 4a,b, the arrows point out the grain boundaries, which give rise to a columnar grain microstructure, or from another point of view, a flat grain microstructure. 

The grey-scale contrasted laths in the micrographs of Figure 4 are structural domains well-known as martensite variants, a distinctive microstructural feature of an SMA in the martensite phase; the contrast is associated with the crystalline orientation of the individual martensites from the self-accommodation group [3,4]. These martensite variants are generally of film thickness and are confined by grain boundaries that span across the thickness of the film. The martensite variants become self-accommodated to minimize stresses from the austenite phase, when the martensite is thermally induced during cooling, giving place to a quasi-zero net macroscopic shape change. This capability produces characteristic self-accommodating variants with a diamond shape, in which martensite could grow in a twin relationship, like those seen in Figure 4c. The identification of the crystalline lattice of these martensites was further established by TEM. These results show that the solid-state diffusion treatment performed on the multilayer system results in a homogeneous Cu-Al-Ni thin film in the austenite phase, with a characteristic columnar grain microstructure, which is further transformed in martensite during quenching.

**Figure 5:**
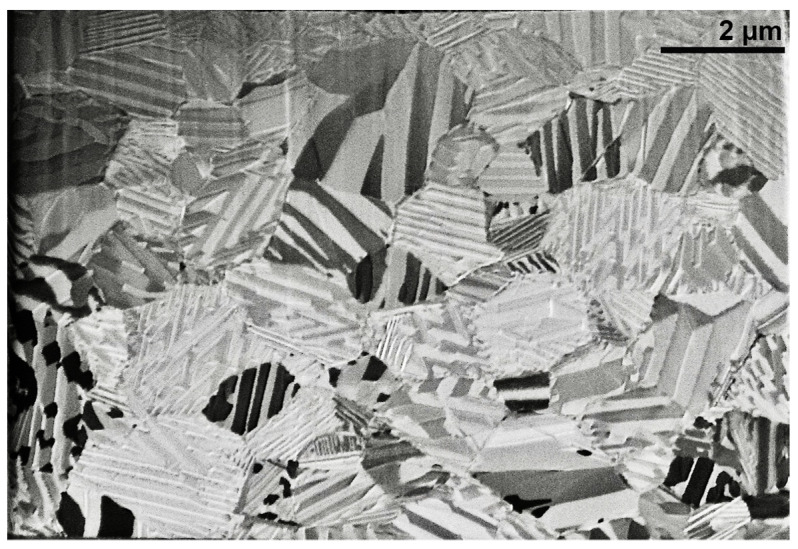
Focused Ga ions image taken on the FIB lamella, revealing the in-plane microstructure of the sample CAN/SN.

Figure 5 is the FIB Ga ion image of a lamella perpendicular to the cross-section that reveals the in-plane thin film microstructure and allows TEM phase characterization and in situ heating. This micrograph confirms the widespread presence of martensite variants within flat grains, as already observed in the cross-sectional microstructure in Figure 4. This kind of microstructure could be favourable for the active superelastic and shape memory properties of this Cu-based SMA. When grain boundaries span across the sample thickness, its energy becomes reduced, as was already shown for unconstrained or monocrystalline 3D microstructures [44,45,46]. Indeed, several studies have shown that the oligocrystalline state—when the surface area is larger than the grain boundary area—is a microstructure that improves the thermomechanical performance in Cu-based SMA polycrystals [53,54,55]. However, this subject is outside the scope of the present work.

**Figure 6:**
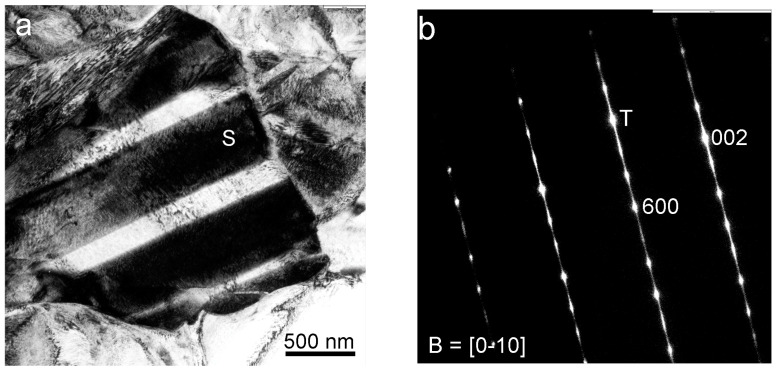
(**a**) BF TEM (200 kV) micrograph of an in-plane grain; (**b**) associated SADP of the variant region labelled S, indexed as β’_3_ martensite (T, transmitted beam).

The martensite phase was identified and characterized by electron diffraction. Figure 6a presents a bright-field (BF) TEM image of a grain with clearly contrasted variants. Figure 6b shows the selected area diffraction pattern (SADP) obtained from the variant labelled with the letter S in the BF image. This diffraction pattern was indexed as the monoclinic β’_3_ martensite (*C2/m*, *a* = 1.38017 nm, *b* = 0.52856 nm, *c* = 0.43987 nm, β = 113.60° [56]), as expected from previous works in similar bulk alloys [50]. The streaks in the diffraction pattern of Figure 6b could be attributed to the presence of many stacking faults inside the martensite variants, probably produced by the internal stresses. 

**Figure 7:**
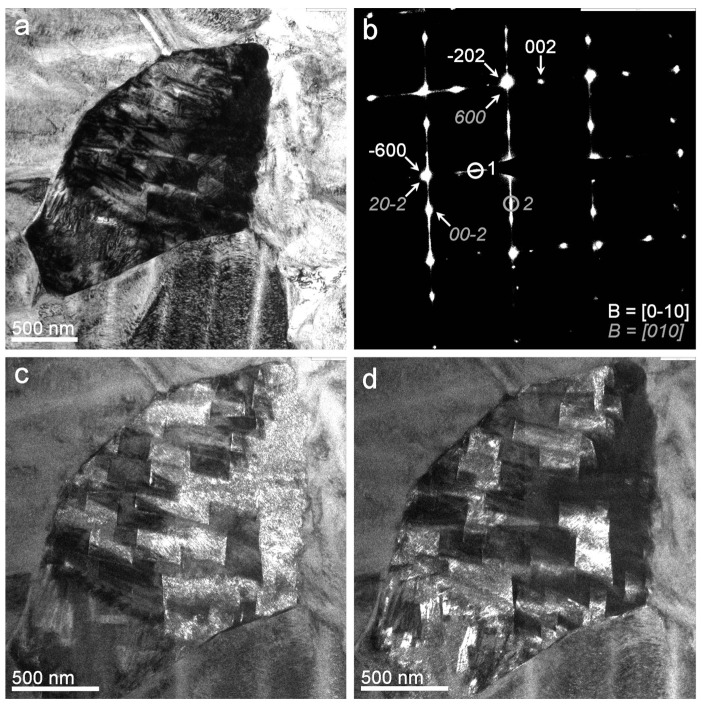
(**a**) BF TEM (200 kV) micrograph of an in-plane grain; (**b**) associated SADP of two twinned β’_3_ martensite variants in the darkness grain; (**c**) dark-field micrograph obtained from spot circle 2 (grey colour); (**d**) dark-field micrograph obtained from spot circle 1 (white colour).

The dominant existence of this phase was confirmed in different grains, as exposed in a representative form in Figure 7, where a twin variant is analysed. The BF TEM image of Figure 7a shows a grain from which the SADP in Figure 7b was taken. This SADP was also indexed as β’_3_ martensite of two variants in a twin relationship.

The dark-field images in Figure 7c,d were obtained from the two spots indicated on the SADP of Figure 7b (surrounded by two small circles coloured white and grey, respectively) to contrast the twinning condition. As can be seen, both images have inverted contrasts inside the grain; that is, the bright zones in the dark-field image of spot one (Figure 7d) are dark in the dark-field image of spot two (Figure 7c) and vice versa. These results prove that the exposed experimental procedure obtained a Cu-Al-Ni thin film in β’_3_ martensite phase.

### 3.2. In Situ TEM Heating Experiment

**Figure 8:**
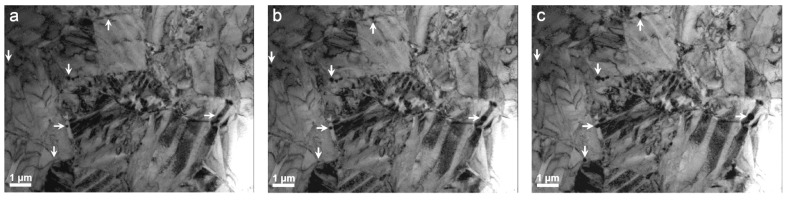
Precipitation observed by TEM (200 kV) in situ heating. BF images (**a**–**c**) were taken at 290, 350, and 450 °C, respectively. The white arrows indicate some nucleated precipitates that grew during heating.

**Figure 9:**
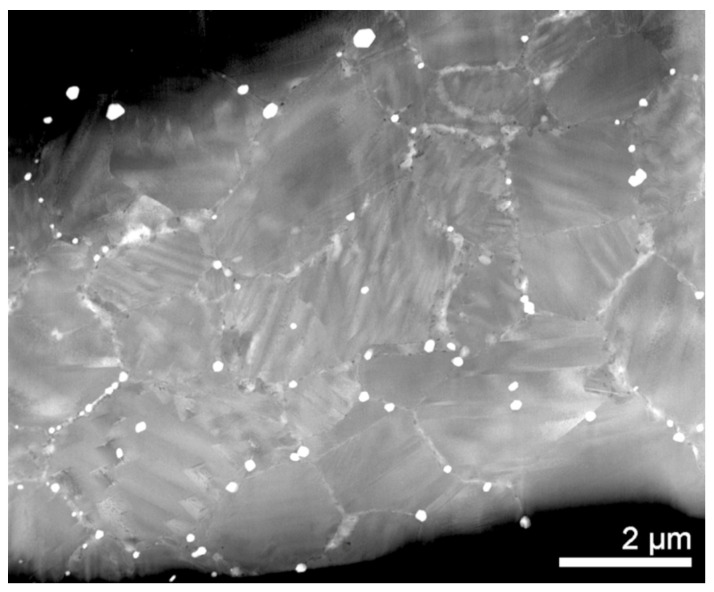
HAADF–STEM image at 300 kV of the sample CAN/SN after in situ heating up to 450 °C, revealing precipitates at grain boundaries and triple junctions.

**Figure 10:**
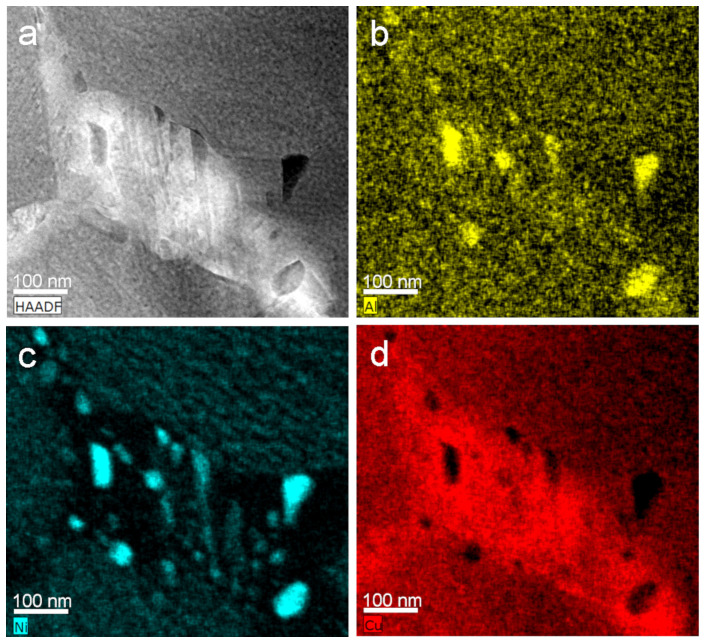
STEM at 300 kV. (**a**) HAADF image; EDX-elemental maps; (**b**) Al (yellow); (**c**) Ni (blue); (**d**) Cu (red).

The in situ heating TEM experiment was carried out in two steps up to a maximum temperature of 450 °C and registered with a video camera. The first heating step proceeded from room temperature to 280 °C at a rate of 5 °C/min as the live sample image was observed. During this step, an active thermal process was observed through the movements of the contrasting waves inside the grains in the 230–260 °C temperature range, which was understood to indicate the martensite transformation into austenite. However, when the thermal program reached the temperature of 280 °C, a rigorous sample examination was performed, and no evidence of the existence of the austenite phase was found—the wave’s movement probably occurred due to the internal stress release. Indeed, an incipient presence of precipitates was visually detected, and the second heating run promoted a coarsening of these precipitates, allowing verification of their nature. The second heating step proceeded from 280 °C to 450 °C at the mentioned rate, and was also observed in the live image, showing that the incipient precipitates became coarse during heating. This coarsening process is illustrated in the three TEM BF images of a fixed area in Figure 8a–c, taken at 290, 350 and 450 °C, respectively. Six of the multiple precipitates in this area have been selected and marked with arrows to demonstrate their increase in size. The BF images during in situ heating were acquired under 200 kV TEM with a rapid-acquisition rate camera, but with high-enough resolution to follow the growth of the precipitates; for instance, the precipitate indicated by the left horizontal arrow grew from about 110 nm to 240 nm, and the one indicated by the left vertical arrow grew from 125 nm to 220 nm, in Figure 8a and Figure 8c, respectively. Clear evidence of the presence of precipitates was observed during this second heating run at 290 °C. After the in situ experiment up to 450 °C, the sample was cooled to room temperature and transferred for observation under 300 kV Titan FEI. Figure 9 shows a HAADF–STEM image revealing the widespread presence of precipitates in grain boundaries and triple junctions. In the HAADF contrast, two types of precipitates appeared: some bright and coarse (>100 nm), as in Figure 9, and others dark and tiny (<100 nm), as in Figure 10. Their chemical nature was analysed by EDX.

Figure 10a shows the HAADF–STEM image, including some dark and grey precipitates, and Figure 10b–d correspond to the mapping composition for Al (yellow), Ni (blue), and Cu (red), respectively. According to the maps, the dark precipitates were rich in Al and Ni and poorer in Cu. This seems to indicate that NiAl-rich intermetallic was formed during the process, or that eventually they corresponded to the equilibrium gamma phase, which was also CuNi-Al-rich, with a much higher content of Cu than the NiAl intermetallic, in agreement with the observed precipitation of stable phases at a macroscopic scale [57], taking place according the ternary phase diagram [52]. The white region in the HAADF image corresponded to a triple point, which became Cu-rich as a consequence of the precipitation. The quantitative analysis of these precipitates was not possible because they were smaller than the sample thickness. On the contrary, the quantitative analysis of the matrix offered a value of 12.6 Al (wt.%), in good agreement with the previously measured Al concentration of the sample.

**Figure 11:**
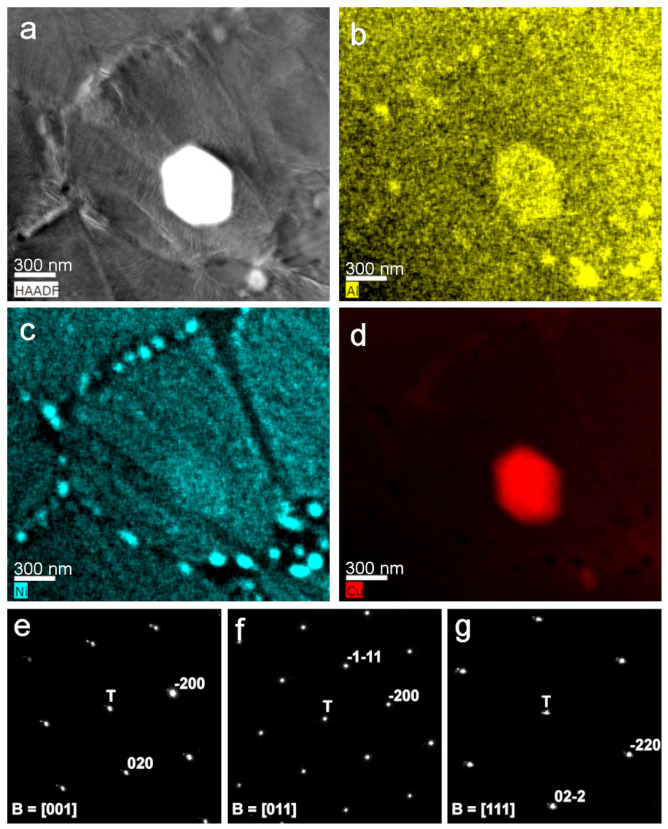
TEM/STEM at 300 kV. (**a**) HAADF image showing the bright precipitate; EDX-elemental maps; (**b**) Al (yellow); (**c**) Ni (blue); (**d**) Cu (red); (**e**–**g**) parallel nanodiffraction patterns (T, transmitted beam) from the bright precipitate, which is indexed as alpha phase.

The HAADF–STEM image of Figure 11a shows a bright precipitate corresponding to one of those whose growth was observed by in situ heating, and also reveals tiny dark grey precipitates, as in the previous Figure 10. Figure 11b–d correspond to the mapping composition for Al (yellow), Ni (blue), and Cu (red), respectively. Once again, the EDX maps show that the nanometric precipitates at the grain boundaries were Ni-Al-rich, corresponding to the same intermetallic observed in Figure 10. In contrast, the bright precipitate was clearly Cu-rich, as in Figure 11d, and the quantitative analysis gave a composition of 93.1Cu-4.8Al-2.0Ni, which probably corresponded to the equilibrium alpha phase. To verify this point, Figure 11e–g show the parallel nanodiffraction patterns on this precipitate for three different zone axes, which were indexed as Cu-rich alpha phase (*Fm-3m*, *a* = 0.3608 nm [52]). From the in situ and post-mortem TEM results, it can be concluded that the observed precipitates corresponded to the expected equilibrium phases, which preferentially nucleated at the grain boundaries, as in Figure 9, but not, or scarcely, at the martensite interfaces.

To close the discussion section, we have to emphasize that the martensite distribution across the thickness and in-plane of the thin film, shown in Figure 4 and Figure 5, respectively, show a homogeneous concentration of the thin film. These results and the microanalysis of Figure 3 validate the employed processing methodology.

The in situ heating experiments under TEM show two different stages of the microstructures’ evolution. First, between 230 and 260 °C, the motion of the contrast waves can be associated with the release of the internal stresses. Indeed, significant internal stresses developed during the thin film production; see [58] for an overview on these stresses. In addition, the further martensitic transformation increased the internal stresses at the grain boundaries because of the high elastic anisotropy of these alloys [3,4]. However, at the surface of the lamella, the stresses relaxed, as occurs in micropillars, causing a delay in the reverse martensitic transformation, because stored internal stresses constitute the driving force for the reverse transformation [46]. This delay in the reverse transformation temperatures was also observed by adiabatic calorimetry [59] and neutron diffraction [60] in bulk Cu-Al-Ni SMA when the stresses were thermally released. This effect of stress relaxation is particularly relevant at the micro- and nanoscale [46], and could have been responsible for the delay in the reverse martensitic transformation during the in situ TEM experiment. Second, for prolonged exposure at 280 °C and above, the nucleation and growth of the precipitates of stable phases, α, γ and NiAl, take place by diffusion processes, preferentially at the grain boundaries. During the first stage (230 to 260 °C), the martensitic transformation is more reproducible and stable, whereas the second one (above 280 °C) promotes the degradation of the microstructure and the loss of shape memory properties. This study demonstrates that thin films of Cu-Al-Ni SMA could satisfactorily work up to the temperature range 230–260 °C. Obviously, for practical applications, the concentration of the alloy must be designed to undergo the martensitic transformation below those temperatures. In addition, the effect of the internal stress relaxation should be quantitatively evaluated in order to match the required transformation temperatures, particularly in microscale thin films. The present work shows that such a design can be achieved through the proposed multilayer processing methodology.

## 4. Conclusions

In the light of the presented results, the following conclusions can be drawn:

- Silicon wafers coated with SiN_x_ (200 nm) constitute a good substrate to grow thin films of Cu-Al-Ni SMA.
- The e-beam evaporation technique of a multilayer stack of Cu, Al, and Ni obtains a homogeneous thin film through further solid-state diffusion treatments.
- The composition of the SMA can be chosen and controlled through the relative thickness of the elemental layers.
- This methodology obtains a homogeneous martensitic transformation along the whole thin film.
- The in situ TEM experiments show that the developed Cu-Al-Ni thin films are thermally stable up to temperatures in the range 230–260 °C, making them very useful as high-temperature shape memory alloys for active applications at the micro- and nanoscales.

These findings show that the used production method obtains Cu-Al-Ni HTSMA thin films with high thermal stability, paving the road for developing high-temperature MEMS/NEMS using shape memory and superelastic technologies.

## Figures and Tables

**Table 1:** Nominal thickness of each elemental metallic layer according to the nominal composition of the Cu-Al-Ni thin film.

Metallic Layer	Nominal Thickness (Å)	Nominal Comp. (wt.%)
Al	1160	11.0
Ni	132/66 *	4.1
Cu	1354	84.9

* Ni layer at the top and bottom.

## Data Availability

The data presented along this work formed part of an ongoing project and are available upon request by e-mail to the corresponding author.
